# A New Promising Anti-Infective Agent Inhibits Biofilm Growth by Targeting Simultaneously a Conserved RNA Function That Controls Multiple Genes

**DOI:** 10.3390/antibiotics10010041

**Published:** 2021-01-04

**Authors:** Thorsten M. Seyler, Christina Moore, Haein Kim, Sheetal Ramachandran, Paul F. Agris

**Affiliations:** 1 Department of Orthopaedic Surgery, Duke University School of Medicine, Durham, NC 277010, USA; christina.moore1@duke.edu; 2 Department of Medicine, Duke University School of Medicine, Durham, NC 27710, USA; haein.kim@duke.edu (H.K.); sheetal.ramachandran@duke.edu (S.R.)

**Keywords:** biofilm, MRSA, RNA target, novel anti-infective tRNA, tRNA-dependent gene regulation

## Abstract

Combating single and multi-drug-resistant infections in the form of biofilms is an immediate challenge. The challenge is to discover innovative targets and develop novel chemistries that combat biofilms and drug-resistant organisms, and thwart emergence of future resistant strains. An ideal novel target would control multiple genes, and can be inhibited by a single compound. We previously demonstrated success against *Staphylococcus aureus* biofilms by targeting the tRNA-dependent regulated T-box genes, not present in the human host. Present in Gram-positive bacteria, T-box genes attenuate transcription with a riboswitch-like element that regulates the expression of aminoacyl-tRNA synthetases and amino acid metabolism genes required for cell viability. PKZ18, the parent of a family of compounds selected in silico from 305,000 molecules, inhibits the function of the conserved T-box regulatory element and thus blocks growth of antibiotic-resistant *S. aureus* in biofilms. The PKZ18 analog PKZ18-22 was 10-fold more potent than vancomycin in inhibiting growth of *S. aureus* in biofilms. In addition, PKZ18-22 has a synergistic effect with existing antibiotics, e.g., gentamicin and rifampin. PKZ18-22 inhibits the T-box regulatory mechanism, halts the transcription of vital genes, and results in cell death. These effects are independent of the growth state, planktonic or biofilm, of the bacteria, and could inhibit emergent strains.

## 1. Introduction

Microbial biofilm formation and homeostasis constitute a major virulence factor in human infections [1]. Biofilm-associated infections are a leading cause of morbidity and mortality in hospitalized patients [2,3]. The prevalence of Gram-positive bacterial, biofilm-associated infections has increased due to the extensive use of medical implant devices [4]. Device surfaces become colonized with Gram-positive microbes that propagate and mature into a biofilm, an immobile, sessile microbial community encased in a protective, self-produced extracellular polymeric matrix (EPM) [5]. Compared to free-floating planktonic organisms, biofilms have the characteristics of a shared physical barrier, rapid intercellular communication, and biofilm-inducible virulence factors that are employed to withstand host stress responses [6,7]. This defensive EPM infrastructure, combined with a slowed metabolism, minimal replication, and emergence of multi-drug resistant bacteria, make biofilm-associated infections notoriously difficult to eradicate [8]. Bacteria growing in a biofilm can evade the host immune system and are up to 1000-fold more resistant to antibiotic therapy compared to their planktonic counterparts [9,10]. Gram-positive bacteria such as *Staphylococcus aureus* and streptococcal species are the most common microbes identified with biofilm-associated infections such as periprosthetic joint infections (70–80% of PJIs) [11,12]. To combat these infections, antibiotic treatment is frequently combined with surgical intervention. Some of the most commonly used antibiotics to treat these infections are vancomycin and gentamicin [13]. In a recent study identifying the common pathogens and drug-resistance rates, 87% of the causative organisms were Gram-positive and vancomycin was the ideal antibiotic for treatment. [14] These findings were corroborated in a study identifying methicillin-sensitive *Staphylococcus aureus* (MSSA), methicillin-resistant *Staphylococcus epidermidis* (MRSE), methicillin-resistant *Staphylococcus aureus* (MRSA), coagulase-negative *Staphylococcus* (CoNS), *Streptococcus,* and *Enterococcus* spp. as major pathogens mainly responsible for implant associated infections [15]. Biofilms on metal implants can be eradicated with vancomycin and tobramycin loaded on calcium beads [16]. The antibiotic loaded beads resulted in an approximate six log reduction and the combination of antibiotic beads and irrigation completely eradicated viable biofilm bacteria. The clinically used antibiotics either inhibit cell wall biosynthesis, protein, or DNA or RNA syntheses, or hinder critical metabolic syntheses [17]. More recently, riboswitches, the regulatory RNA elements mainly located within the 5′UTR of mRNAs, have received increased attention. The targeting of a singular regulatory RNA element, such as a riboswitch, is a novel approach and effective for combating drug-resistant bacteria and biofilm-associated infections [18,19]. Analogs of a metabolite can lethally affect expression of a single essential gene [18,19,20,21], e.g., an inhibitor of the riboflavin riboswitch has proven to be a viable drug candidate [22,23,24]. Within two years of demonstrating riboflavin analogs could inhibit the riboflavin riboswitch, strains resistant to the inhibitors have already emerged [25,26,27,28]. Other resistant strains have also developed riboswitches with constitutive gene expression increasing the pathogen’s viability [25,26,27,28]. In cultures, 5-(3-(4-fluorophenyl)butyl)-7,8-dimethylpyrido[3,4-b]quinoxaline-1,3(2*H*,5*H*)-dione (5FDQD) has potent and rapid bactericidal activity against *Clostridium difficile* and has a low frequency of resistance (<1 × 10^−9^). In C57BL/6 mice, 5FDQD completely prevented the onset of lethal antibiotic-induced *C. difficile* infection (CDI) [29]. The riboswitch has been demonstrated as a sensor of metabolism; thereby small compounds could be discovered to obstruct essential cell processes [30]. Inhibitors of bacterial aminoacyl-tRNA synthetases (aaRS) have been proven effective [31], as have small-molecule inhibitors of RNA functions [32]. Oligonucleotide hybridization to rRNA (akin to antisense) has been suggested as another strategy to prevent resistance [33], however sequence selectivity and access to sequestered biofilm sequences seems to be problematic. Disabling biofilm resistance requires identifying unique targets and developing therapeutics that effectively disrupt critical pathways in biofilm formation, homeostasis, and the emergence of resistance.

In the case of *S. aureus*, resistance has surfaced through a variety of mechanisms such as enzymatic inactivation, altered drug binding affinities, antibiotic trapping, efflux pumps, acquisition of antibiotic resistant gene cassettes (*mec* antibiotic resistant elements), or spontaneous mutation with positive selection—in response to the exposure of each new antibiotic [34]. Biofilms provide additional problems, as reduced antibiotic susceptibility is due to a different combination of factors, including poor antibiotic penetration, reduced growth-dependent killing, altered microenvironments (e.g., pH, low oxygen concentration), adaptive responses, and the formation of persister cells [8,34,35,36]. New therapeutic strategies exploiting multiple mechanisms are urgently needed to combat biofilm-associated infections and emerging resistance most often caused by Gram-positive bacteria [37,38]. The discovery of small molecules or enzymes and unique targets is needed to address the challenge of the physical biofilm barrier or to be incorporated into novel coatings to modify biomaterials used in the medical devices industry, preventing implant devices from being colonized by antibiotic-resistant biofilms [39,40].

**Figure 1:**
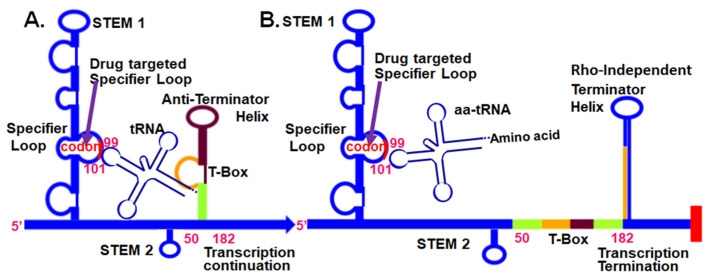
tRNA-dependent, T-Box, regulatory mechanism at the 5’UTR of aminoacyl-tRNA synthetase mRNAs in Gram-positive bacteria. (**A**) Unacylated tRNA bound to the T-box 5′UTR through the anticodon and the 3′-terminal CCA sequence. (**B**) The aminoacylated tRNA does not bind stably to the 5′UTR. Small-molecule intervention (arrow) that inhibits Specifier Loop codon interaction with an unacylated (or acylated) tRNA’s anticodon will terminate transcription [41].

The research reported here responds to the critical need to characterize unique RNA targets for novel compounds that inhibit RNA function in biofilms. We hypothesize that similar to planktonic cultures, biofilm growth would be susceptible to a single novel anti-infective compound that binds specifically to the bacteria and inhibits multiple genes. Previously, we reported a family of small molecules shown to selectively inhibit Gram-positive bacterial growth by preventing the functions of similar regulatory elements, the tRNA-dependent control of gene expression or T-box genes. The T-box regulatory elements control transcription of multiple genes of critical “housekeeping” proteins. The parent of the family of compounds that prevent T-box function was designated PKZ18, 3-((4-(4-Isopropylphenyl)-5-methylthiazol-2-yl)carbamoyl)bicyclo[2.2.1]heptane-2-carboxylic acid (Supplementary Figure S1) [41]. PKZ18 was shown to be non-toxic in eukaryotic cells in culture at the minimum inhibitory concentration (MIC) for methicillin-resistant *Staphylococcus aureus* (MRSA). Prior research demonstrated that eight of 12 of the T-box gene functions are inhibited in *Staphylococcus aureus* [42]. Here we report for the first time a derivative of PKZ18, PKZ18-22 (3-[[[4-[4-(2-Methylpropyl)phenyl]-2-thiazolyl]amino]carbonyl]bicyclo[2.2.1]heptane-2-carboxylic acid), inhibits biofilm growth more comprehensively than clinically-used antibiotics, and works synergistically with common antibiotics. The T-box mechanism exhibits a number of characteristics that are advantageous to drug development against biofilms. T-box function is similar to riboswitches in that a ligand, in this case an unacylated tRNA, binds to a nascent mRNA leader sequence, 5′UTR, and induces a conformational change from an intrinsic Rho-independent transcription terminator helix to a competing transcription anti-terminator helix (Figure 1) [43,44]. Formation of the anti-terminator facilitates full transcription of the downstream gene(s) (Figure 1A) [45]. We demonstrated that initial binding of the tRNA to its cognate regulatory sequence occurs through canonical base pairing of the anticodon and a complementary “codon” in the Specifier Loop of Stem 1 of the nascent mRNA 5′UTR (Figure 1A) [33,41,42]. Unacylated tRNA stabilizes the formation of an anti-terminator hairpin with other base-pairings, chiefly between the tRNA’s conserved, unacylated universal 3′-terminus (5’-NCCA-3’) and a 7-nucleotide bulge (5′-UGGN-3′) in the anti-terminator hairpin [33,43,45]. Aminoacylated tRNA is unable to bind the 7-nucleotide bulge and stabilize the anti-terminator conformation, resulting in terminator helix formation and transcription termination (Figure 1B) [43,44,45,46]. Thus, inhibiting the primary base pair formation between the tRNA anticodon and the Specifier Loop codon (Figure 1) effectively reduces transcription of essential genes [41]. We have previously shown that PKZ18 analogs acted synergistically in planktonic cultures with aminoglycosides to significantly enhance the efficacy of the analogs and aminoglycosides, further increasing their therapeutic windows [42]. The research presented here is specifically about the interactions of PKZ18-22 with biofilms. We demonstrate the breadth of this putative antibiotic in inhibiting biofilms with superior potency than commonly clinically used antibiotics. Here, we conclude that PKZ18-22 is 10-fold more active than vancomycin against *S. aureus* and acts synergistically with gentamicin.

## 2. Results

Our previous work [41,42] supports the premise that a unique RNA function regulating many genes in Gram-positive bacteria can be specifically targeted and inhibited by small molecules.

### 2.1. Biofilm Growth Is Retarded by PKZ18 Analogs

**Figure 2:**
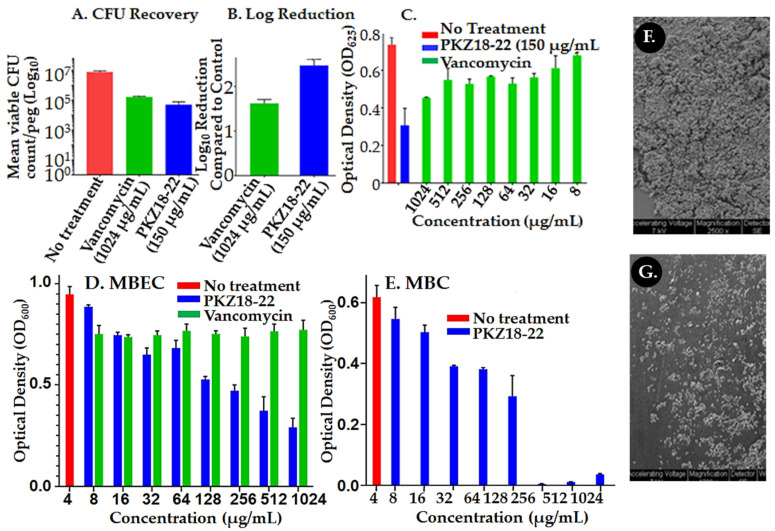
Assessment of PKZ18-22 effect on *Staphylococcus* biofilms. (**A**) *S. aureus* biofilm colony forming units (CFU/log_10_) after 24 h of a single dose antibiotic and the PKZ18 analog PKZ18-22. (**B**) Log reduction after a single dose 24 h challenge. PKZ18-22 treatment exhibited 2.5 log reduction, which corresponds to >99% bacterial cell reduction; significantly greater reduction compared to vancomycin. (**C)** Growth of *S. aureus* after 24 h exposure to PKZ18-22 vs. dosages of vancomycin in the minimum biofilm eradication (MBEC) biofilm model. Optical density measured as a surrogate for turbidity/bacterial growth demonstrated significant growth reduction with PKZ18-22 treatment compared to all vancomycin concentrations and negative controls. (**D**) The MBEC efficacy of a range of concentrations of PKZ18-22 compared with vancomycin using the same parameters as C. (inoculum, media concentration, etc.) but using new lot of PKZ18-22 and vancomycin stocks. No growth with vancomycin. (**E**) The minimum bactericidal concentration (MBC) efficacy of a range of concentrations of PKZ18-22. Scanning electron micrographs of peg surfaces: (**F**) no treatment and (**G**) treatment with PKZ18-22.

PKZ18-22 activity against known biofilm-producing methicillin-resistant *Staphylococcus aureus* (MRSA) was evaluated using an established MBEC^TM^-HTP biofilm model (Innovotech) for studies of implant-associated infections [47]. PKZ18-22 (150 μg/mL) demonstrated superior potency when compared to vancomycin (1024 μg/mL). Vancomycin has long been considered an antibiotic of last resort against Gram-positive antibiotic-resistant bacteria and has been shown to be most valuable in treating PJIs [14]. It is a glycopeptide and hinders bacterial growth by inhibiting peptidoglycan cross linkage during bacterial cell wall synthesis. Resistance emerges by the bacterium substituting an amino acid in a cell-wall component, preventing vancomycin from binding. The mean recovery growth of the PKZ18-22-treated *S. aureus* was 4.3 log_10_ CFU/mL versus 5.2 log_10_ CFU/mL for the vancomycin-treated pegs on which the biofilms were grown (*p* = 0.01; Figure 2A). This corresponded to a 2.5 log reduction in CFU/mL for the PKZ18-22-treated biofilms compared to 1.6 for the vancomycin-treated samples (Figure 2). After 24 h of exposure, it was observed that PKZ18-22 at 150 μg/mL had the capacity to eradicate biofilms more efficiently than any of the vancomycin concentrations tested (Figure 2B,C). We compared the minimum biofilm eradication concentrations (MBEC) efficacy over a range of concentrations for PKZ18-22 and vancomycin against *Staphylococcus* biofilms (same parameters as Figure 2C: inoculum, media concentration, etc., except plates read at 600 instead of 625) (Figure 2D). The vancomycin concentrations showed less efficacy compared to PKZ18-22. The highest concentration of vancomycin was not effective. We checked the efficacy over a range of concentrations for PKZ18-22 and vancomycin against planktonic growth of *Staphylococcus*. There was no growth with vancomycin; PKZ18-22 exhibited a killing curve. Using scanning electron microscopy, we saw that the PKZ18-22-treated biofilms differed in cell morphology, thickness, and population density compared with the untreated biofilms (Figure 2F,G).

### 2.2. Synergistic Activity of PKZ18-22 with Common Clinical Antibiotics

**Figure 3:**
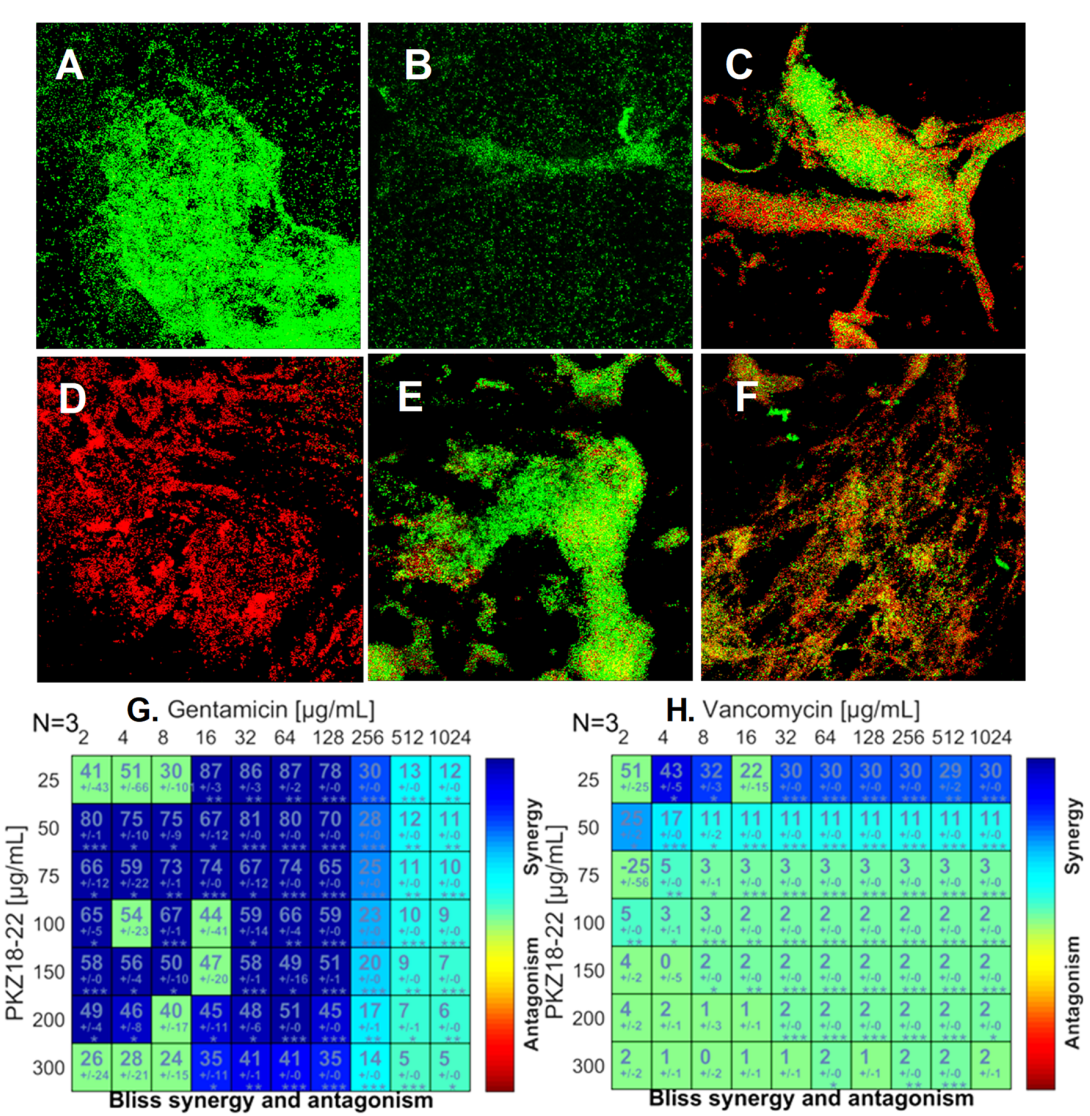
PKZ18-22–antibiotic synergy on *S. aureus* biofilm growth. (**A**) Biofilm grown for 24 h with no treatment. (**B**) Biofilm growth after 24 h exposure to 25 µg/mL PKZ18-22. (**C**) Growth after 24 h exposure to 4 µg/mL gentamicin. (**D**) Growth after 24 h exposure to 64 µg/mL gentamicin + 25 µg/mL PKZ18-22. (**E**) Growth after 24 h exposure to 4 µg/mL vancomycin. (**F**) Growth of *S. aureus* after 24 h exposure to 4 µg/mL vancomycin + 25 µg/mL PKZ18-22. (**G**) Synergy score matrix for drug combination of PKZ18-22 and gentamicin using a drug interaction Bliss reference model. The highest drug synergy was observed with PKZ18-22 of 25 µg/mL and gentamicin concentrations of 16–64 µg/mL (*p* < 0.001). (**H**) Synergy score matrix for drug combination of PKZ18-22 and vancomycin. By definition all synergy scores above zero are synergistic. Synergy scores of PKZ18-22 and vancomycin exhibited significantly lower effects compared to PKZ18-22 and gentamicin. The highest synergy score was observed at 4 µg/mL vancomycin + 25 µg/mL PKZ18-22. Asterisk’s indicate level of confidence in results, probability of obtaining test results: * indicates *p* < 0.05; ** indicates *p* < 0.001; *** indicates *p* < 0.0001.

The Bliss independence model was used to analyze the activity of PKZ18-22 in combination with established antibiotics such as gentamicin, rifampin, and minocycline. The combination of PKZ18-22 (25 μg/mL) and gentamicin (64 μg/mL) demonstrated superior potency against a MRSA (ATCC 29213) biofilm when compared with each using an established MBEC™-HTP biofilm model (Figure 3). Synergistic combinations with a measured score higher than 25 were classified as positive. The highest synergy score of 87 was achieved using a combination of PKZ18-22 (25 μg/mL) and gentamicin (16 μg/mL and 64 μg/mL). These highly synergistic effects were not observed in any of the PKZ18-22 and vancomycin combinations, which reached a maximal synergy score of only 43 (PKZ18-22, 25 μg/mL and vancomycin, 4 μg/mL).

**Figure 4:**
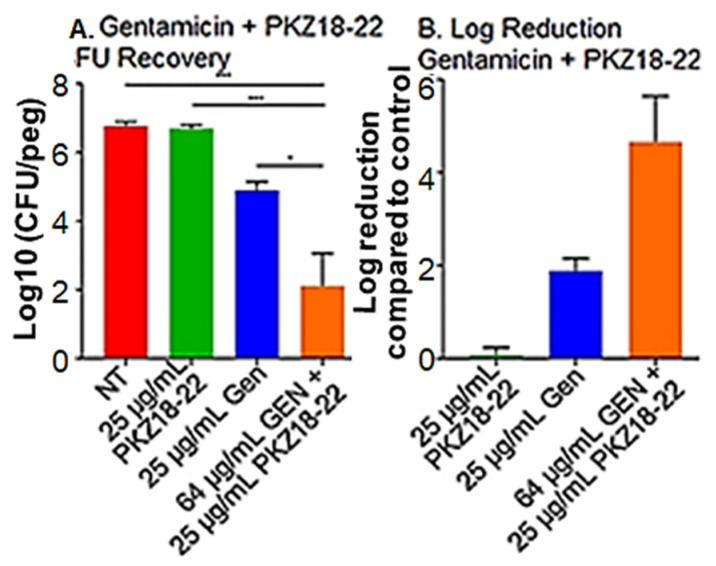
PKZ18-22 plus gentamicin inhibition of biofilms. (**A**) *S. aureus* biofilm CFU/log10 after 24 h of a single dose gentamicin, PKZ18-22, and the combination of gentamicin and PKZ18-22. The following asterisk values using a Student’s *t*-test indicate: * indicates *p* < 0.05; *** indicates *p* < 0.001. (**B**) After a single dose 24 h challenge, the combination of gentamicin/PKZ18-22 resulted in a cell reduction of ~5 logs, >99.9%. A significantly greater reduction when compared to each treatment alone.

The most promising combination of PKZ18-22 (25 μg/mL) and gentamicin (64 μg/mL) produced an almost 5 log reduction in CFU/mL in biofilm growth after 24 h exposure (Figure 4). Similar results noted with PKZ18-22 and rifampin (Supplementary Figure S2). PKZ18-22 concentrations at 25 and 50 μg/mL showed the highest synergy. These highly synergistic effects were not observed in any of the PKZ18-22 and vancomycin combinations. The highest synergy, with a score of 43, was noted with a combination of PKZ18-22 (25 μg/mL) and vancomycin (4 μg/mL). When focusing on the most promising combination of PKZ18-22 (25 μg/mL) and gentamicin (64 μg/mL), there was an almost ~5 log reduction after 24 h exposure. The combination of PKZ18-22 with gentamicin exhibited a greater clinical potential to treat methicillin-susceptible *S. aureus* biofilms when compared to any of the PKZ18-22 and vancomycin tested concentrations. After a single dose 24 h challenge, the combination of minocycline and PKZ18-22 did not produce a significantly greater reduction when compared to each treatment alone. Thus, PKZ18-22 was not synergistic with minocycline.

### 2.3. Synergy of PKZ18 and Common Antibiotics in Planktonic Cultures

The combinatorial strategy to improve efficacy is already in wide clinical use [48,49,50,51]. Previously, we found that the T-box function could be targeted using improved PKZ18 analogs alone and in novel synergistic combinations with aminoglycoside antibiotics on planktonic cultures, increasing the efficacy of both by 4–8-fold [41,42]. PKZ18-22 and other analogs 18-52, and 18-53 showed synergistic activity with gentamicin. We were encouraged with these results demonstrating synergy with planktonic cultures (unpublished at the time) to test whether antibiotics would be synergistic with PKA18-22 against biofilms (Figure 3 and Supplemental Figure S2).

## 3. Discussion

Gram-positive bacterial biofilms have “outsmarted” both researchers and clinicians. In developing effective antibacterial compounds against biofilms, we must overcome the protective nature of the growth characteristics and extracellular polymeric matrix (EPM) of the biofilm. In addition, the bacteria evolve antibiotic resistance to the drug target. Thus, the difficulty in finding a new small-molecule drug to penetrate the biofilm and be effective in inhibiting growth and preventing the emergence of resistance is essentially a quest to discover the “Achilles heel” of the organism. Researchers are continuing to look for the one target that would satisfy the biofilm properties, yet they find, as with planktonic cultures, resistance evolving from target alteration, efflux of the antibiotic and enzyme-catalyzed chemical modification, and amino group acetylation. In the history of antibiotic resistance, researchers have encountered bacteria’s ability to defeat the designed drugs to kill them by the emergence of resistant strains. Emergence of resistance has become ever more rapid as a new antibiotic is introduced, due to the new chemical entity being similar to the older version, and its binding to targets similar to the older version. Pharmaceutical companies are reluctant to invest in discovery and development due to the high cost of research and low margins.

We have introduced a new and effective strategy of finding novel putative antibiotics that abrogate resistance and the emergence of new resistant strains [42]. As we found in support of our hypothesis with planktonic growth, biofilm growth is susceptible to a single novel anti-infective compound that binds specifically to a set of similar RNAs that control multiple genes. This compound is capable of penetrating, in effective concentrations, the EPM. tRNA-dependent control of gene expression, the T-box, is a unique target that could be exploited in the development of small-molecule antibacterial biofilm therapies against the most virulent and drug-resistant Gram-positive pathogens. The T-box is found in Gram-positive pathogens but not in Gram-negative bacteria with exception of the d-proteobacteria [52] and not found in the human host [52,53]. In terms of our hypothesis, the T-box leader sequence fulfills the criteria of a promising drug target for combating biofilm-associated infections [41,42] (Figure 1) that is phylogenetically conserved and exclusive to Gram-positive organisms. Inhibition of the T-box regulatory mechanism halts the expression of aminoacyl-tRNA synthetase genes and genes involved in amino acid metabolism and transcription, ultimately resulting in cell death. This effect is independent of the bacteria’s growth state. PKZ18-22 is found to be effective against biofilms and thwarts drug resistance [41,42]. PKZ18-22 binds to the conserved structural element of the T-box Specifier Loop and prevents the codon–anticodon interaction required for tRNA binding. It could possibly deform the structure and disrupt the function of multiple Specifier Loops concurrently, repressing a considerable number of essential genes, e.g., 12 conserved T-box regulons for *S. aureus*, 7–10 for *Streptococci* spp., 20 in *C. difficile*, and 39 in *B. anthracis* [52]. Simultaneous mutations in T-box sequences that confer resistance would be highly improbable. Likewise, mutations of a number of tRNA anticodons that bind the Specifier Loop codons are unlikely to evolve; they are recognition determinants of many aaRSs and crucial for decoding in translation.

T-box function has been studied by us [33,41,42] and others previously. Researchers showed that the anti-terminator can be targeted with substituted oxazolididones [54,55,56,57,58]. However, the anti-terminator T-box sequence, UGGN, is common to RNAs that bind NCCA, the 3′-terminus of tRNA. Therefore, specificity is lost and many off-site events would occur. We demonstrated that small-molecule drugs may be highly suitable for targeting the T-box mechanism in biofilms (Figure 2). PKZ18-22 selectively targets Gram-positive bacteria, directly reduces transcriptional read-through, and is refractory to resistance [41,42]. The minimum inhibitory concentration (MIC) of PKZ18-22 against most bacteria was 8–32 µg/mL [41,42]. However, it inhibits biofilm growth synergistically with gentamicin. As such, PKZ18-22 is an important proof of principle showing that small-molecule antibiotics against biofilms are suitable for targeting similar multiple RNA mechanisms, but further development is required.

## 4. Materials and Methods

### 4.1. Determining if Biofilm Formation Is Susceptible to PKZ18 Analogs In Vitro

Biofilm formation starts from planktonic bacteria, followed by adhesion to an organic or abiotic surface. Following the initial adhesion, sessile microcolonies form and extracellular polymeric substance (EPS) is secreted. Bacteria embedded in EPS often enter a “stationary-like” phase or persister status, making them less susceptible to conventional antibiotics that target growing cells. An overnight culture of *S. aureus* (ATCC 29213) was diluted to 1 × 10^7^ cells/mL in tryptic soy broth (TSB) supplemented with 10% human plasma (Innovative Research, Novi, MI USA) and added to the wells of an MBEC^TM^-HTP Assay Biofilm Innoculator (Innovotech, Edmonton, AB, Canada). The plate was incubated overnight at 37 °C and shaken at 125 rpm. After 24 h of growth the lid of the plate was removed, rinsed with PBS, and transferred to a standard 96-well plate containing dilutions of PKZ18-22 and vancomycin prepared in TSB. Control wells were without a PKZ18 analog. The treatment plate was incubated for 24 h at 37 °C after which the lid was removed, rinsed with PBS, and placed in a new 96-well plate containing TSB. The biofilm was removed from the assay lid into the recovery plate wells by sonication, a new plate cover was added, and the viability of the biofilm was determined after 24 h of incubation at 37 °C by reading the OD at 625 nm. Three independent experiments were conducted. Concentrations of interest were followed up with colony forming unit (CFU) recovery and scanning electron microscopy analysis. After conclusion of the MBEC assay, the minimum bactericidal concentration (MBC) was determined. The MBC is the lowest concentration of an antibacterial agent required to kill a particular bacterium. The peg lid from the MBEC assay was removed from the challenge plate and 20 μL of medium from each well of the challenge plate was removed and added to a new sterile 96-well plate filled with 180 µL TSB. The new 96-well plate was then covered with a regular lid and allowed to incubate for 24 h before MBC values were determined using an automated plate reader to obtain optical density measurements at 600 nm (OD_600_).

### 4.2. Determine the Synergy of Different PKZ18 Analogs and Antibiotic Combinations to Treat Established Staphylococcus Biofilms In Vitro

Bacteria growing in a biofilm matrix are intrinsically more resistant to environmental agents and have been shown to tolerate antibiotic concentrations 10- to 1000-fold higher than the corresponding planktonic counterpart. This inherent resistance of bacteria in biofilms and the emergence of antibiotic-resistant bacteria has necessitated the drive to explore competent novel antimicrobial agents such as PKZ18 and the development of combinations of these novel antimicrobial agents with established antibiotics. We determined the minimum biofilm eradication concentration (MBEC) of PKZ18 compounds in combination with vancomycin, gentamicin, rifampin, and tetracycline. Three independent experiments were conducted and required a checkerboard dilution to test various concentrations of the following combinations (PKZ18 analog + each antibiotic) against *S. aureus* utilizing an established MBEC™-HTP biofilm model (Innovotech) [59,60,61].

Briefly, an overnight culture of *S. aureus* was diluted to 1 × 10^5^ cells/mL in cation-adjusted Mueller Hinton broth (MHB) supplemented with 1% human plasma and added to the wells of an MBEC plate. The plate was incubated overnight at 37 °C and shaken at 125 rpm. Following biofilm growth, the lid was then transferred to a standard 96-well plate in which dilutions of PKZ18-22 and the designated antibiotics were prepared individually and in combination in MHB. The treatment plate was incubated for 24 h at 37 °C. After incubation, the lid was removed and rinsed in PBS. Following treatment, a tetrazolium (2,3,5-triphenyl tetrazolium chloride, TTC) assay was performed to assess metabolic activity and viability. The lid was rinsed in PBS, transferred to a 96-well plate containing a 0.01% TTC solution in MHB, and incubated at 37 °C overnight. To dissolve the stain from the pegs, the lid was placed in a 96-well plate containing 96% ethanol and the OD of the wells was then read at 490 nm. Data was analyzed with the Bliss independence model using the Combenefit software [62]. The most promising combinations were further investigated with CFU recovery and confocal microscopy analysis.

### 4.3. CFU Recovery

After rinsing in PBS, designated pegs were snapped off the lid using sterile tweezers and placed in 1.5 mL Eppendorf tubes containing 200 µL PBS. Biofilm was disrupted from the pegs by sonicating the tubes for 15 min (Branson M8800H, Branson Ultrasonics, West Chester, PA, USA), followed by vortexing for 10 s. Three independent serial dilutions were prepared for each sample and plated on tryptic soy agar (TSA) plates that were incubated overnight at 37 °C. The plates were then imaged and colonies counted on a ColonyDoc-It™ Imaging station (UVP, Analytik Jena, Beverly, MA, USA).

### 4.4. Scanning Electron Microscopy

Pegs designated for scanning electron microscopy were snapped off the lid using sterile tweezers and fixed with a 2.5% glutaraldehye solution (MilliporeSigma, St. Louis, MO, USA) in 0.2 M sodium cacodylate buffer, pH 7.4 (Electron Microscopy Sciences, Hatfield, PA, USA) for at least 24 h at 4 °C. The pegs were then removed from the fixative and rinsed in cacodylate buffer. The pegs were post-fixed for 1 h with 2% osmium tetroxide (Ted Pella, Inc., Redding, CA, USA) and then washed again with cacodylate buffer. The pegs were dehydrated in increasing concentrations of ethanol (30%, 50%, 70%, 90%, 100%) and then allowed to air dry overnight. After drying, the pegs were mounted on stubs and sputter coated with gold using a Denton Desk V vacuum sputter system and imaged on a FEI XL30 scanning electron microscope.

### 4.5. Confocal Laser Scanning Imaging

Pegs designated for confocal microscopy were snapped off the lid using sterile tweezers and stained with a SYTO 9/propidium iodide (LIVE/DEAD, BacLight; Invitrogen, Waltham, MA, USA) solution. The pegs were incubated, covered from the light, for 20 min. After incubation, the pegs were rinsed in PBS and placed on 50 mm glass-bottom dishes (MatTek, Ashland, MA, USA). The pegs were imaged using a Leica SP5 Inverted Confocal Microscope (Leica Microsystems, Buffalo Grove, IL, USA) at a resolution of 512 × 512 pixels using a 63× water immersion objective (63×/1.2W).

## 5. Conclusions

Small molecules bind non-coding RNAs and structure/function base assays can be developed to select these molecules [63,64]. A new class of antibacterial reagent is a novel hybrid small molecule but has yet to be shown for long-term resistance or active against biofilms [65]. We conclude that the compound PKZ18-22 is a very effective antibiotic against *S. aureus* biofilms. It penetrates the extracellular polymeric matrix, has a reasonable MIC for an initial construct, and has the potential to prevent the emergence of drug resistant strains. PKZ18-22 and gentamicin proved synergistic activity against both *S. aureus* biofilm and planktonic cultures.

## Data Availability

The data that support the findings of this study are available from the corresponding authors (T.M.S. or P.F.A.), upon reasonable request.

## Supplementary Materials

The following is available online at https://www.mdpi.com/2079-6382/10/1/41/s1, Figure S1: Chemical structures of PKZ18 and analog PKZ18-22, Figure S2: Synergistic activity of PKZ18-22 with rifampin.
