# Supplementary material for: A New Promising Anti-Infective Agent Inhibits Biofilm Growth by Targeting Simultaneously a Conserved RNA Function That Controls Multiple Genes
Source: Antibiotics (Basel). 2021 Jan 4;10(1):41. doi: 10.3390/antibiotics10010041 (PMC7824582; doi:10.3390/antibiotics10010041)
Supplement: Supplementary file 1 [file antibiotics-10-00041-s001.pdf]

Supplementary Materials

A New Promising Anti-Infective Agent Inhibits Biofilm Growth by Targeting Simultaneously a Conserved RNA Function that Controls Multiple Genes

Thorsten M. Seyler [1,*], Christina Moore [1], Haein Kim [2], Sheetal Ramachandran [2] and Paul F. Agris [2,*]

[1] Department of Orthopaedic Surgery, Duke University School of Medicine, Durham, NC 277010, USA; christina.moore1@duke.edu

[2] Department of Medicine, Duke University School of Medicine, Durham, NC 27710, USA; haein.kim@duke.edu (H.K.); sheetal.ramachandran@duke.edu (S.R.)

* Correspondence: thorsten.seyler@duke.edu (T.M.S.); paul.agris@duke.edu (P.F.A.); Tel.: +919-613-7797 (T.M.S.); +919-681-1218 (P.F.A.)

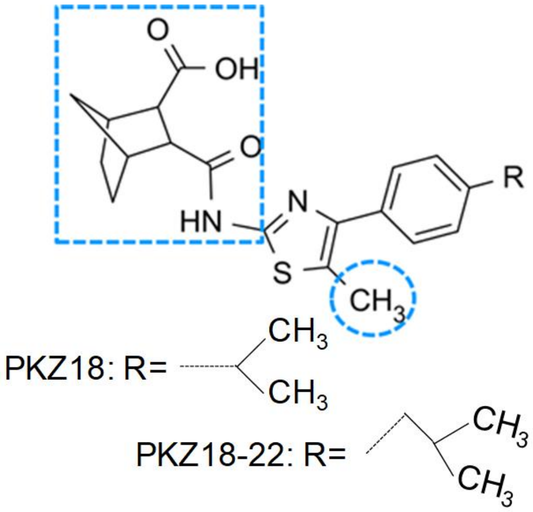

Figure S1. Chemical structures of PKZ18 and analog PKZ18-22. Features identified by structure activity relationships as increasing activity (PKZ18 isobutyl substitute for PKZ18-22 isopropyl). The norbornane is necessary for Gram-positive specificity (dashed box); the methyl is not necessary for activity (dashed circle). (United States Patent 10,266,527.).

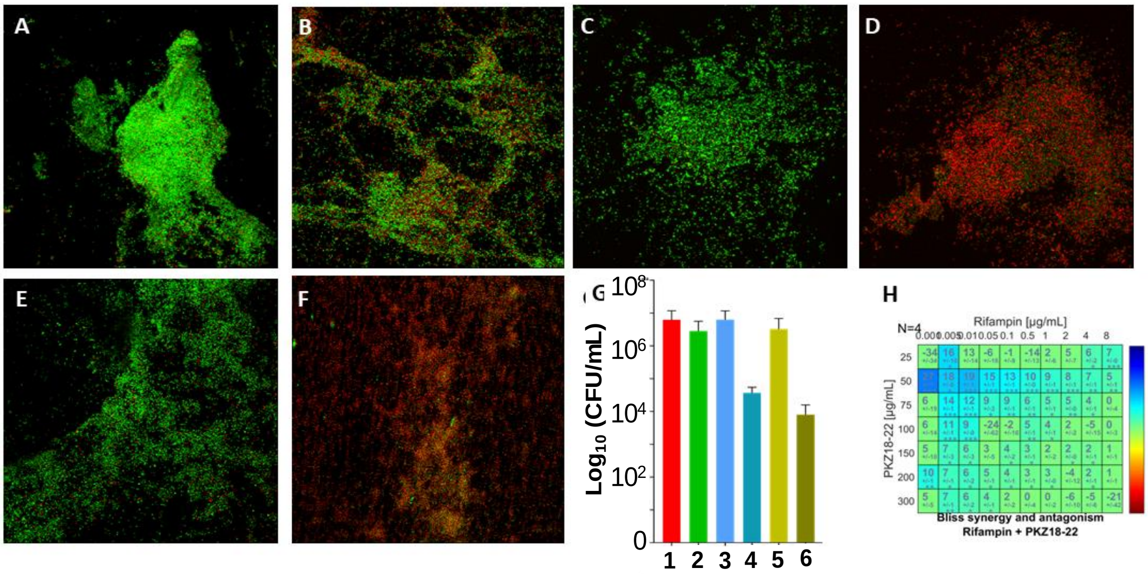

Figure S2. Synergistic activity of PKZ18-22 with rifampin. A. Biofilm grown for 24 h with no treatment. **B.** Biofilm growth after 24 h exposure to 25 µg/mL PKZ18-22. **C.** Growth after 24 h exposure to 0.001 µg/mL rifampin. **D.** Growth after 24 h exposure to 0.001 µg/mL rifampin + 25 µg/mL PKZ18-22. **E.** Growth after 24 h exposure to 0.005 µg/mL rifampin. **F**. Growth after 24 h exposure to 0.005 µg/mL rifampin + 25 µg/mL PKZ18-22. **G.** 1, red *S. aureus* biofilm colony forming units (CFU/log_{10}) growth not treated; 2 green, after 24 h of a single dose PKZ18-22 (25 µg/mL); 3, blue 0.001 µg/mL rifampin; 4, teal, 0.001 µg/mL rifampin and PKZ18-22 (25 µg/mL); 5, mustard 0.005 µg/mL rifampin; 6, brown, 0.005 µg/mL rifampin and PKZ18-22 (25 µg/mL). After single dose 24 h challenge, the combination of rifampin and PKZ18-22 produced a significant greater reduction when compared to each treatment alone. **H.** Synergy score matrix for drug combination of PKZ18-22 and rifampin using a drug interaction Bliss model. Highest drug synergy observed with PKZ18-22 of 50 µg/mL and rifampin concentrations of 0.001–0.005 µg/mL ($p < 0.001$).
